# A Low Affinity GCaMP3 Variant (GCaMPer) for Imaging the Endoplasmic Reticulum Calcium Store

**DOI:** 10.1371/journal.pone.0139273

**Published:** 2015-10-09

**Authors:** Mark J. Henderson, Heather A. Baldwin, Christopher A. Werley, Stefano Boccardo, Leslie R. Whitaker, Xiaokang Yan, Graham T. Holt, Eric R. Schreiter, Loren L. Looger, Adam E. Cohen, Douglas S. Kim, Brandon K. Harvey

**Affiliations:** 1 National Institute on Drug Abuse, National Institutes of Health, 251 Bayview Blvd, Baltimore, Maryland, 21224, United States of America; 2 Department of Chemistry and Chemical Biology, Harvard University, Cambridge, Massachusetts, 02138, United States of America; 3 Department of Physics, Harvard University, Cambridge, Massachusetts, 02138, United States of America; 4 Harvard Stem Cell Institute, Harvard University, Cambridge, Massachusetts, 02138, United States of America; 5 Howard Hughes Medical Institute, Harvard University, Cambridge, Massachusetts, 02138, United States of America; 6 Janelia Research Campus, Howard Hughes Medical Institute, 19700 Helix Drive, Ashburn, Virginia, 20147, United States of America; Cinvestav-IPN, MEXICO

## Abstract

Endoplasmic reticulum calcium homeostasis is critical for cellular functions and is disrupted in diverse pathologies including neurodegeneration and cardiovascular disease. Owing to the high concentration of calcium within the ER, studying this subcellular compartment requires tools that are optimized for these conditions. To develop a single-fluorophore genetically encoded calcium indicator for this organelle, we targeted a low affinity variant of GCaMP3 to the ER lumen (GCaMPer (10.19)). A set of viral vectors was constructed to express GCaMPer in human neuroblastoma cells, rat primary cortical neurons, and human induced pluripotent stem cell-derived cardiomyocytes. We observed dynamic changes in GCaMPer (10.19) fluorescence in response to pharmacologic manipulations of the ER calcium store. Additionally, periodic calcium efflux from the ER was observed during spontaneous beating of cardiomyocytes. GCaMPer (10.19) has utility in imaging ER calcium in living cells and providing insight into luminal calcium dynamics under physiologic and pathologic states.

## Introduction

Calcium ions are important for a wide array of biological processes, including proper functioning of the endoplasmic reticulum (ER). Calcium within the ER lumen is maintained at levels 5000 times greater than the cytoplasm (~500 μM versus ~100 nM) using an energy intensive process where calcium ions are pumped up a concentration gradient by the sarco/endoplasmic reticulum calcium ATPase (SERCA) pump [1, 2]. Efflux from the ER is mediated by calcium channels, primarily the ryanodine (RyR) and IP_3_ (IP_3_R) receptors, which open in response to cellular signals[3].

Tight control over ER calcium is necessary for functions intrinsic to the ER, including protein folding, protein trafficking, and lipid synthesis [4, 5]. Additionally, the gradient created across the ER lipid bilayer is critical for many signaling pathways [6]. Some ER-calcium-dependent processes are ubiquitous across cell types, such as activity of the calcium-binding chaperones BiP, GRP94, and calreticulin [7]. Other functions are more cell-type specific. For example, in neurons, the ER serves as the source of intracellular calcium that regulates neurotransmitter release [8], synaptic plasticity[9], neuronal growth [10], and communication between dendrites and the nucleus [11]. Dysregulation of ER calcium has been proposed to contribute to several neuronal pathologies including Alzheimer’s Disease [12, 13], Parkinson’s Disease [14], and Huntington’s Disease [15, 16]. In cardiac muscle, calcium storage within the sarcoplasmic reticulum (SR) is essential for muscle contractility, and impaired uptake of calcium into this organelle is a common characteristic of heart failure [17].

Investigating ER calcium dynamics presents a technical challenge due to the high concentration of ions in this compartment. Calcium flux into the cytoplasm (probed using genetically encoded sensors or calcium sensitive dyes) is often used as a proxy for the ER calcium content [18, 19]. However, cytoplasmic measurements can be affected by many factors, such as store operated calcium entry [20], activity of voltage gated calcium channels [21], rate of mitochondrial uptake[22], or rate of extrusion into the extracellular space [23]. For these reasons, methods to visualize directly the ER calcium store, such as low affinity calcium dyes, have been utilized [24]. Calcium dyes, however, lack subcellular targeting information and often require removal of cytosolic dye using detergents that permeabilize the plasma membrane [25]. Enhanced dye localization can be achieved by coupling an AM-ester form of a low affinity calcium dye with transgenic expression of an ER-localized esterase [26].

Genetically encoded calcium indicators (GECIs) can incorporate subcellular targeting information, but many of these proteins have calcium affinities too high for use in the ER. Several ER-specific GECIs have been developed, including the commonly used FRET-based D1ER [27]. While D1ER has been enormously valuable in the study of ER calcium, this sensor has a limited dynamic range and requires specialized microscopic equipment. Aqueorin-based calcium sensors, which utilize the substrate coelenterazine, have also been optimized for the ER environment [5, 28]. Recently, several groups have reported non-ratiometric fluorescent ER calcium indicators [29–31]. A study by Suzuki and colleagues [29] modified GECO color variants [32] to contain a mutated calcium-binding calmodulin domain, such that the calcium affinity was reduced to match calcium concentrations in the ER. These multi-colored ER calcium indicators, which were given the name CEPIA1er (Calcium-measuring organelle-Entrapped Protein IndicAtor 1 in the ER), showed improved dynamic range compared to D1ER [29].

Here, we report the development of an additional low affinity variant of GCaMP, in this case GCaMP3, which we have termed GCaMPer. This variant was identified in a large-scale mutagenesis effort to find GCaMP variants with desirable characteristics [33]. GCaMPer, like the recently reported CEPIA1er, has utility in the studies of subcellular compartments enriched in Ca^2+^. In this study, we report the construction and characterization of a set of virally encoded GCaMPer tools to interrogate ER calcium dynamics in cells of neuronal and cardiac origin.

## Materials and Methods

### Plasmids and cloning

The low affinity GCaMP3 variant (10.19; D324G, D360G, D397G, D435G) was identified in a previous screen for improved GCaMP calcium indicators [33]. Both the WT and 10.19 versions of GCaMP3 were targeted to the ER by fusing the calreticulin signal peptide (MLLSVPLLLGLLGLAVA) to the amino terminus of GCaMP3. The signal peptide is predicted to be cleaved co-translationally [34]. The ER retention signal from BiP (TAEKDEL) was fused to the carboxy terminus. To create the fusions, an intermediate pAAV-EF1α plasmid was created in which the calreticulin signal peptide and TAEKDEL sequences were flanked by BspQI sites (to create scarless cloning sites). This intermediate was created by inserting the BspQI-flanked N- and C-terminal targeting sequences (synthesized as a gBlock DNA fragment; Integrated DNA Technologies) into the BamHI and EcoRI sites of pOTTC374 (pAAV-EF1α double-floxed iRFP, AddgenePlasmid #47626). The intermediate plasmid was then linearized with BspQI and combined with PCR products encoding the GCaMP3 variants. The GCaMP3 variants were PCR-amplified with the following InFusion-compatible (Clontech) primers: 5’-ggcctggccgtcgccatgggttctcatcatcatcatcatcatg-3’ and 5’-atctttttctgctgtcttcgctgtcatcatttgtacaaactct-3’. For human synapsin 1 promoter (hSYN1) GCaMPer constructs, the coding sequence was subcloned into pOTTC427 (pAAV-hSYN1-eGFP) using the BamHI and EcoRI sites (replacing eGFP with GCaMPer). All clones were transformed and propagated in Stbl3 recombination-deficient competent cells (Life Technologies) and sequence verified. The lentiviral expression plasmid was created by subcloning GCaMPer into the FCK lentiviral expression plasmid [35] under control of a CMV promoter using a BamHI/EcoRI restriction digest. The constructs described in this manuscript have been deposited in the Addgene plasmid repository. See S1 Fig for plasmid schematics and Addgene numbers. Addgene ID numbers (in parentheses) are as follows: pAAV-EF1α-GCaMPer (10.19) (# 63885), pAAV-EF1α-GCaMPer (GCaMP3) (# 63884), pAAV-hSYN1-GCaMPer (10.19) (# 63887), pAAV-hSYN1-GCaMPer (GCaMP3) (#63886), and pLenti-CMV-CRTsigpep-GCaMP3(10.19)-KDEL (#65227).

### Cell culture

SH-SY5Y human neuroblastoma cell lines cells were maintained in DMEM (4.5 g/L glucose, 110 mg/mL sodium pyruvate) supplemented with 10% bovine growth serum (Hyclone), 10 units/mL penicillin, and 10 μg/mL streptomycin. Cells were grown in a humidified incubator at 37°C with 5.5% CO_2_. For transient transfections, SH-SY5Y cells were transfected with 0.25 μg DNA and 0.53 μL Lipofectamine 2000 (prepared in OptiMem) per cm^2^. Complexes were removed from the cells after 4 h and replaced with complete media. Rat primary cortical neurons (PCNs) were prepared as described previously [36] and in accordance with approved procedures by the NIH Animal Care and Usage Committee. PCNs were cultured in Neurobasal medium (Life Technologies) supplemented with 1X B27 (Life Technologies) and 0.5 mM L-glutamine. Cells were plated at 6x10^4^ viable cells per well in polyethyleneimine-coated 96-well plates. Cells were fed by 50% media exchange starting on the 4th day *in vitro* (DIV4). Additional feedings were conducted on DIV6, 8, 11, and 13. Human induced pluripotent stem cell-derived cardiomyocytes (hiPSC-CMs) were obtained from a commercial source (CDI, iCell cardiomyocytes). Cells were cultured on 35-mm glass bottom dishes (MatTek Corp., P35G-1.5-7-C) pre-coated with fibronectin (Yo Proteins, 663). Cells were plated following CDI’s protocols at a density of 50,000 living cells per cm^2^, and cultured in CDI maintenance medium. Lentiviral infection was performed 4 days after plating and cells were imaged 14 days after plating. Cells were imaged in a home-made DMEM medium without phenol red: the formulation matched Life Technologies #11054, but without amino acids or vitamins. Additionally, to prevent metabolic shock and ensure cell beating during imaging, glucose was replaced with 10 mM galactose [37].

### Virus (AAV and Lenti) production and titering

AAV virus was produced as previously described [38]. Briefly, virus was packaged in HEK293 cells by the triple transfection method [36]and purified on an AVB sepharose HI-TRAP column (GE Healthcare) using an AKTA purifier (GE Healthcare). Virus was titered by digital droplet PCR (Bio-Rad) using the hSYN1 promoter as the target sequence. Briefly, the AAV was diluted 10^−5^ and 10^−6^ using PBS. Five microliters of diluted vectors was added to 20 μL of master mix (final concentrations: 1X ddPCRSupermix for Probes (No dUTP) (Bio-Rad), 450 nM forward primer (sequence: gccctgcgtatgagtgcaagtg), 450 nM reverse primer (sequence: ggatgcgcaatttggggaatgg), 50 nM probe (sequence: accgaccccgacccactggacaagcac), and water. PCR mixes were placed in the automated droplet generator (ADG, Bio-Rad) for droplets. The droplets were run through a thermal cycler program to completion and read using a digital droplet PCR reader (Bio-Rad). Viral titers are reported as viral genomes/mL. Low titer lentivirus was produced in HEK293T cells with the following protocol: cells were plated at a density of 5.2 x 10^4^ cells per cm^2^ in 35 mm plastic dishes. Twenty-four hours after plating the medium was partly aspirated to leave 1 mL per dish, and the DNA mix was prepared by adding, in sequence, 250 μL of OptiMem, 0.9 μg of GCaMPer (10.19) vector, 0.9 μg of psPAX2 packaging vector and 0.6 μg of VsVg envelope vector. Lastly, 7.5 μL of TransIT®-293 reagent (Mirus Bio LLC) was added and, after a rest period of ~15 min at room temperature, the DNA mixture was added drop wise to the cells. Thirty-six hours after transfection the supernatant was collected, filtered with a 0.45 μm syringe filter, aliquoted and either added to the hiPSC-CMs (250 L per 35 mm dish) or stored at -80°C for later use.

### Immunocytochemistry

SH-SY5Y cells were seeded at 4.0x10^5^ cells in 35 mm dishes containing 25 mm round glass coverslips (Warner Instruments) and allowed to adhere overnight. Cells were transfected with the GCaMPer plasmid DNA using 2 μL Lipofectamine 2000 (Invitrogen) and 0.4 μg DNA per well. Forty hours after transfection, cells were fixed with 4% paraformaldehyde in PBS (pH 7.4), permeabilized with PBS + 0.1% TritonX-100 + 0.2% BSA for 15 min. Permeabilized cells were blocked with PBS + 0.1% TritonX-100 + 5% goat serum for 1 h at room temperature. Primary antibodies (described in antibody reagents section) were prepared in fresh blocking solution, added to cells, and incubated overnight at 4°C. Secondary antibodies (AlexaFluor, Invitrogen) were added for 1 h at room temperature. All washes were done with PBS + 0.1% TritonX-100 at room temperature and nuclei were stained with 1 μg/mL DAPI (in PBS) for 15 min. Coverslips were mounted on glass slides with Mowiol 4–88 (EMD) and allowed to set overnight. A Nikon Eclipse-C1 confocal microscope equipped with a Nikon 100X (1.30 NA) PlanFluor objective was used to image samples. Nikon EZ-C1 software was used for image acquisition. Micrographs were prepared using Adobe Photoshop or ImageJ (NIH).

### In vitro calcium calibration

For calibration of calcium response of purified GCaMP3 (10.19), protein was purified as described [33]. An 11-point calcium titration (100 nM to 10 mM) was performed by diluting a 10 mM CaCl_2_ stock solution in 30 mM MOPS (pH 7.2) and 100 mM KCl and adding purified protein (1:50) in duplicate samples. Green fluorescence (ex: 485 ± 5 nm, em: 510 ± 5 nm) was read with a Safire2 plate reader (Tecan). Sigmoidal binding curves were fit to the data to extract the Hill coefficient and K_d_ for Ca^2+^. Determination of pKa values was carried out by pH titration of purified protein in either 5 mM EGTA or 100 mM CaCl_2_ as described [33]. Magnesium titrations were conducted by diluting purified protein in 10 mM MOPS, pH 7.0, 100 mM KCl with varying MgCl_2_ from 100 μM to 10 mM. Values for k_off_ were determined using a stopped-flow device (Applied Photophysics) coupled to a fluorometer (Varian). Protein samples in 5 mM calcium were rapidly mixed with a solution of 10 mM EGTA. Solutions were buffered with 50 mM MOPS pH 7.2, 100 mM KCl at room temperature. Single exponential curves were fitted to the fluorescence decay.

For calcium calibration of GCaMPer in primary neurons, cells were seeded in polyethyleneimine-coated 96-well plates at 6x10^4^ viable cells per well. Cells were transduced on DIV6 with AAV-hSYN1-GCaMPer (1.9 x 10^9^ vg). One week after transduction, culture medium was replaced with imaging medium containing 20 mM HEPES (pH 7.5), 150 mM NaCl, 5 mM KCl, 1 mM MgCl_2_, 1 mM CaCl_2_, and 5.25 mM D-glucose (BSA was not included as it interferes with ionomycin [39]). Cells were allowed to equilibrate in the new medium for 60 min. Pre-equilibration images were captured and x-y-z field coordinates were recorded using Prior Proscan III motorized stage and Nikon Elements software. Medium was then exchanged with imaging medium (as above, but without calcium) supplemented with 20 μM A23187 (ionophore), 10 μM ionomycin (ionophore), 1 μM thapsigargin (SERCA inhibitor), and the appropriate concentration of Ca^2+^ by adding CaCl_2_. Fluorescence images were captured (from identical fields) after 65 min.

### GCaMPer imaging using flow chambers

For SH-SY5Y experiments, cells were re-plated 48 h post-transfection to an Ibidi μ-Slide VI ^0.4^ at density of 6.0x10^4^ viable cells/cm^2^. After 3 h of cell attachment, the outer reservoirs were filled with culture medium and returned to incubator overnight. Before imaging, culture medium was exchanged with imaging medium containing (20 mM HEPES pH 7.4, 150 mM NaCl, 5 mM KCl, 1 mM MgCl_2_, 10.5 mM D-glucose, and 1.9 mg/mL bovine serum albumin). For rat primary cortical neuron experiments, cells were plated in poly-d-lysine coated Ibidi μ-Slide VI ^0.4^ at 2.0x10^5^ cells per channel (30 μL volume). After 3 hours (to allow cells to adhere within the channel), the outer chambers were filled with PCN feed medium. Medium in the outer chambers was replaced every other day for the duration of the experiment. Cells were transduced on DIV6 with AAV-hSYN1-GCaMPer (10.19) or AAV-hSYN1-GCaMPer (GCaMP3). For transductions, medium was completely removed from the outer chambers (without disturbing the medium in the channel). Fifty microliters of fresh PCN medium containing virus (1.0–2.0 x 10^10^ vg), was added to the outer chamber, and slide was returned to incubator. After 3 h, the outer chambers were filled with fresh PCN medium. For all flow chamber experiments, slide chambers were placed within a 10 cm tissue culture dish containing a 35 mm dish filled with water, to ensure humidified atmosphere and to minimize evaporative effects.

For imaging experiments, temperature of flow solutions was maintained between 27 and 28°C using an Automatic Temp Controller (TC-344B, Warner Instruments, Hamden, CT) and multi-line in-line solution heater (64–0104, Warner Instruments). Flow of solutions was controlled by a Masterflex C/L pump (Cole Parmer, Vernon Hills, IL) attached to a pinch valve assembly (Warner Instruments). Pinch valve positions were controlled using Nikon Elements AR software interfacing with a VC-6 Six Channel Valve Controller (Warner Instruments). The dead-time of the setup (time from switching solution to reaching the chamber slide) was two minutes. The dead-time of the system is accounted for in the presentation of all data.

### Imaging and micrograph analysis for neurons

Live-cell imaging was performed on a Nikon EclipseTE2000-E inverted microscope equipped with a Nikon 20X (0.45 NA) Plan Fluor objective or 60X (1.40 NA, oil immersion) and an Andor iXon3 high sensitivity EMCCD camera (Andor Technology, Belfast, UK). Multi-point x-y-z coordinates were set using a Prior Proscan III motorized stage (Prior Scientific, Rockland, MA). To account for Z-drift, images were captured in five Z-planes (10 micron below initial plane to 10 microns above) with the Elements AR software used to determine the in-focus plane for collection. For fluorescence quantitation (data presented in figures are from single representative experiments), individual cells were selected from multiple fields of view using ImageJ software. Background-subtracted fluorescence intensity was measured at each timepoint (to derive F_t_). Relative fluorescence (F_t_/F_0_) was calculated as a ratio relative to fluorescence at timepoint zero. For experiments where X-Y drift occurred, the TurboReg and StackReg plugins (Philippe Thévenaz) were utilized to align images before analyses.

### Imaging of Cardiomyocytes

For active recording, cells were imaged with a custom, low-magnification microscope with 3 μm resolution and a maximum 6 x 6 mm field of view. The GCaMPer was excited with 490 nm LED, collected through a 540/50 nm bandpass emission filter, and recorded on a Hamamatsu ORCA-Flash 4.0 sCMOS camera. During imaging, cells were maintained at 37°C under humidified air with 5% CO_2_. Perfusion was implemented using a custom-machined flat glass window inserted into 35 mm tissue culture dish, which allowed for fast medium exchange and optical access from above and below the culture. Syringes with imaging buffer and drug were heated to 37°C and bubbled with 5% CO_2_ in air.

### Chemical and antibody reagents

Stock solutions were prepared as follows: thapsigargin (Sigma, 1 mM, DMSO), (S)-3,5-DHPG (Tocris, 20 mM, H_2_O), ionomycin (Sigma, 5 mM, DMSO), A23187 (Sigma, 30 mM, DMSO), 4-chloro-m-cresol (Sigma, 1 M, DMSO), cyclopiazonic acid (Sigma, 25 mM, DMSO), caffeine (Sigma, 5 mM, H_2_O). Antibodies used were chicken polyclonal anti-GFP (Aves # GFP-1020), rabbit monoclonal anti-RCAS1 (Cell signaling # 6960), and mouse monoclonal anti-PDI (Abcam # ab2797). Secondary antibodies were Alexa Fluor 488 and Alexa Fluor 568 (Life Technologies).

## Results

**Fig 1:**
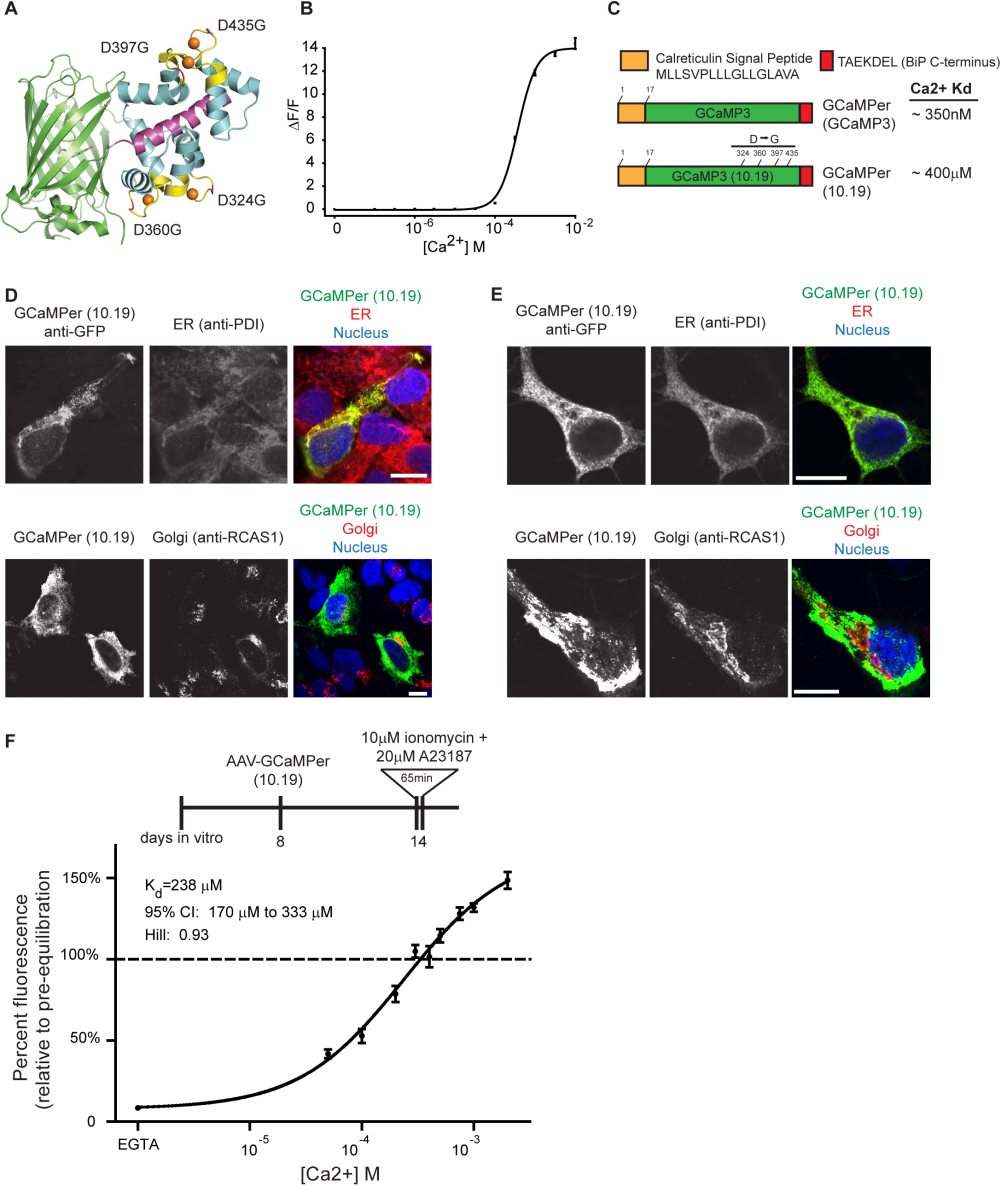
Targeting a low affinity GCaMP3 variant to the endoplasmic reticulum. (a) Mutations to GCaMP3 (10.19 variant) creates a GECI with reduced affinity for calcium [33]. The EF-hand loops (yellow), mutated aspartic acid residues (red), and calcium ions (orange) are indicated on the GCaMP3 structure (PDB: 3SG7). (b) Ca^2+^ titration of purified GCaMP3 variant 10.19. K_d_ for Ca^2+^ ~ 400 μM, Hill slope ~ 1.9, ∆F_max_/F_0_ ~ 14. Points represent ∆F/F_0_ (mean ± range, n = 2). (c) Schematic diagram of ER-targeting approach for GCaMP3 (10.19), creating GCaMPer. *In vitro* Ca^2+^ affinities for the untagged GCaMP3 variants are indicated. (d) Confocal microscopy of GCaMPer (10.19) localization in human neuroblastoma (SH-SY5Y) cells. Cells were transfected with pAAV-EF1α-GCaMPer (10.19 variant) and immunostained using GFP, PDI (ER resident chaperone), and RCAS1 (Golgi protein) antibodies. Nuclei were stained with DAPI. Scale bar = 10 microns. (e) Confocal microscopy of GCaMPer (10.19) localization in rat primary cortical neurons. Cells were transduced with AAV-hSYN1-GCaMPer (10.19) and immunostained using GFP, PDI, and RCAS1 antibodies. Nuclei were stained with DAPI. Scale bar = 10 microns. (f) GCaMPer (10.19) fluorescence in rat primary cortical neurons over a range of calcium concentrations after equilibrating calcium across cellular membranes using ionophores A23187 and ionomycin (mean ± SEM, n = 30 cells).

The GCaMP3 variant (10.19) used to make GCaMPer was identified in a screening effort for novel GECIs. The 10.19 variant has 4 amino acid substitutions in the EF-hand loops that coordinate calcium ions (Fig 1A). These mutations reduced calcium affinity from K_d_ ~ 350 nM [33] to K_d_~ 400 μM, a concentration similar to that found in the endoplasmic reticulum [4] (Fig 1B). The offset of calcium-induced fluorescence was faster for GCaMP3 variant 10.19 (GCaMP3 k_off_: 0.89 s^-1^; GCaMP3 variant 10.19 k_off_: >80 s^-1^). The Hill slope was also reduced from 2.5 [33] to 1.9. Magnesium affinity remained negligible for both GCaMP3 and GCaMP3 variant 10.19 (1.8 mM and 5.8 mM respectively), and maximal responses to magnesium were low (ΔF_max_/F_0_: 0.27 and 0.71, respectively). Incorporation of the 4 mutations in the EF-hand loops did not alter pH sensitivity (GCaMP3 pK_a,apo_ 8.1, pK_a,Ca_ 7.2; GCaMP3 variant 10.19 pK_a,apo_ 8.0, pK_a,Ca_ 7.2).

The low affinity GCaMP3 variant (10.19) coding sequence was fused downstream of a signal peptide from calreticulin to allow access to the secretory pathway. An ER-retention sequence, identical to the final seven amino acids of the ER chaperone GRP78/BiP (TAEKDEL), was fused to the carboxy-terminus. The fusion protein co-localized with the ER chaperone protein disulfide isomerase (PDI) in both SH-SY5Y neuroblastoma cells and rat primary cortical neurons (Fig 1D and 1E; PDI panels). Minimal colocalization with the Golgi apparatus marker RCAS1was observed, indicating a functional ER-retention motif (Fig 1D and 1E; RCAS1 panels). The calcium affinity of GCaMPer (10.19) in rat primary cortical neurons was 238 μM (Hill slope = 0.93), as determined by fluorescence changes following Ca^2+^ equilibration using ionomycin and A23187 (Fig 1F). Based on this calibration curve, basal ER calcium in the rat primary cortical neurons was approximately 335 μM.

**Fig 2:**
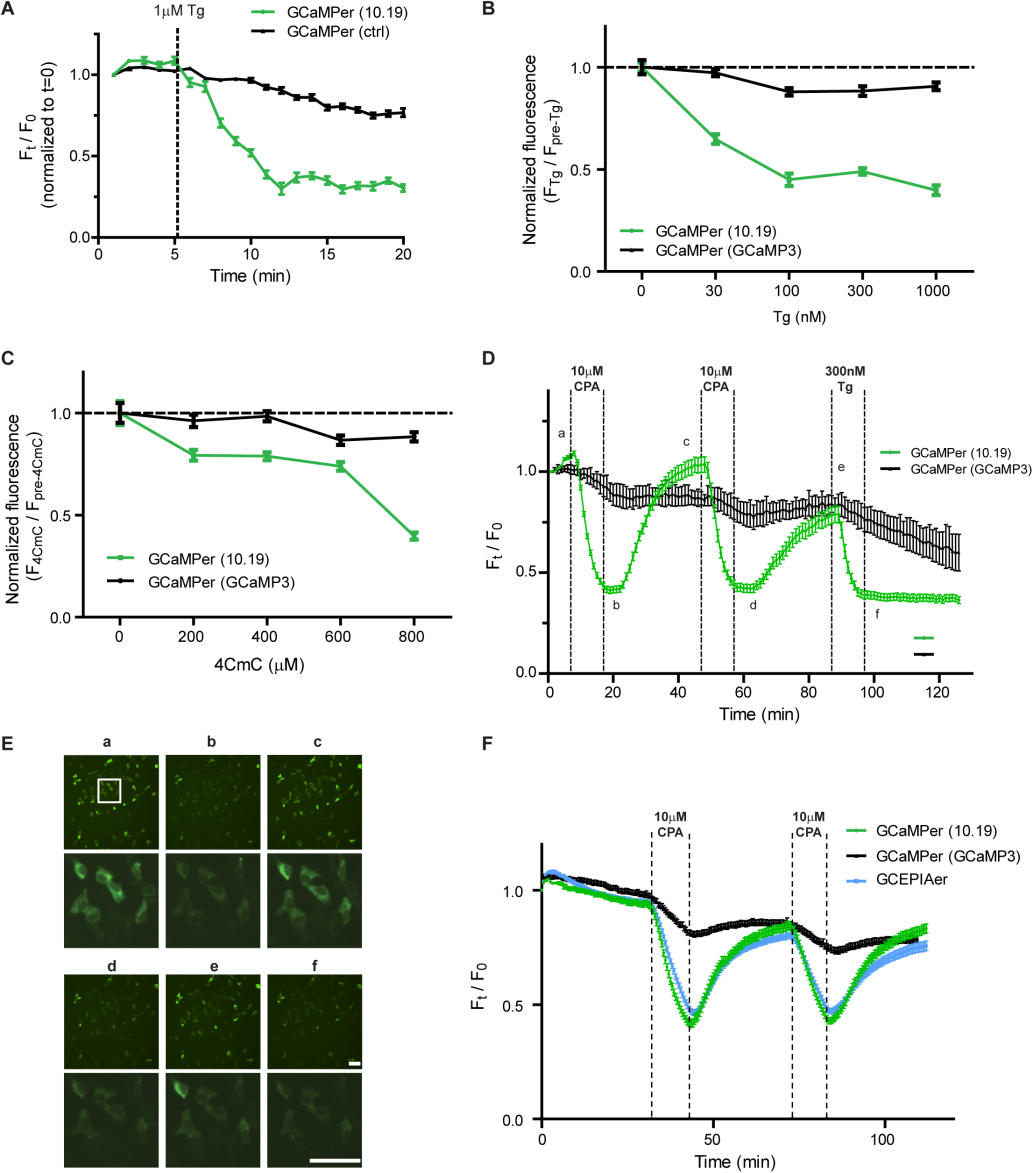
GCaMPer fluorescence monitored in SH-SY5Y cells exposed to pharmacologic modulators of ER calcium. (a) SH-SY5Y cells transfected with GCaMPer (10.19 or GCaMP3) were exposed to 1 μM thapsigargin and imaged over 20 min (mean ± SEM, n = 30 cells per variant). (b) GCaMPer (10.19) fluorescence in response to 30 min of indicated doses of thapsigargin (mean ± SEM, n = 50 cells per variant). (c) GCaMPer (10.19) fluorescence in response to 30 min of indicated doses of 4-chloro-m-cresol, a RyR agonist (mean ± SEM, n = 50 cells per variant). (d) GCaMPer (10.19 or GCaMP3) fluorescence in response to reversible SERCA inhibition with cyclopiazonic acid (CPA). Two cycles of 10 μM CPA addition and washout were performed, followed by thapsigargin to irreversibly inhibit SERCA. Fluorescence of individual cells was tracked over the duration of the experiment (mean ± SEM, n = 100 cells per variant). (e) Micrographs corresponding to data presented in panel e. Scale bars = 100 microns. (f) Comparison of GCaMPer and G-CEPIA1er in SH-SY5Y cells. Cells were equilibrated in imaging medium for 30 min prior to beginning flow and imaging. Two cycles of 10 μM CPA addition and washout were performed and fluorescence of individual cells was tracked over the duration of the experiment (mean ± SEM, n = 100 cells per variant).

The ability of GCaMPer (10.19) to report ER calcium dynamics was first assessed in immortalized SH-SY5Y neuroblastoma cells. As a control for non-specific decreases in fluorescence due to secretion, degradation, or photobleaching of the reporter, we used the high affinity GCaMP3 (GCaMPer (GCaMP3), K_d_~350 nM) targeted to the ER via the same tags used for the low affinity GCaMPer. SH-SY5Y cells expressing GCaMPer were exposed to thapsigargin (Tg), to irreversibly inhibit SERCA, thus depleting the ER calcium store. A decrease in fluorescence was detected for GCaMPer (10.19), with minimal effect on GCaMPer (GCaMP3) (Fig 2A). A dose-response relationship between thapsigargin and GCaMPer (10.19) fluorescence was observed (Fig 2B). As an alternate approach to deplete ER calcium, we employed the ryanodine receptor (RyR) agonist 4-chloro-m-cresol (4CmC), which caused a dose-depended decrease in GCaMPer (10.19) fluorescence (Fig 2C). Next, we assessed recovery of GCaMPer (10.19) fluorescence following refilling of the ER store by using a reversible SERCA inhibitor, cyclopiazonic acid (CPA). GCaMPer (10.19) fluorescence recovered following washout of CPA, over multiple cycles of drug addition and wash-out, suggesting GCaMPer is suitable for examining dynamic changes in ER calcium (Fig 2D and 2E, S1 Video). We next compared GCaMPer (10.19) to the recently reported G-CEPIA1er [29], and observed similar changes in fluorescence in response to CPA treatment (Fig 2F).

**Fig 3:**
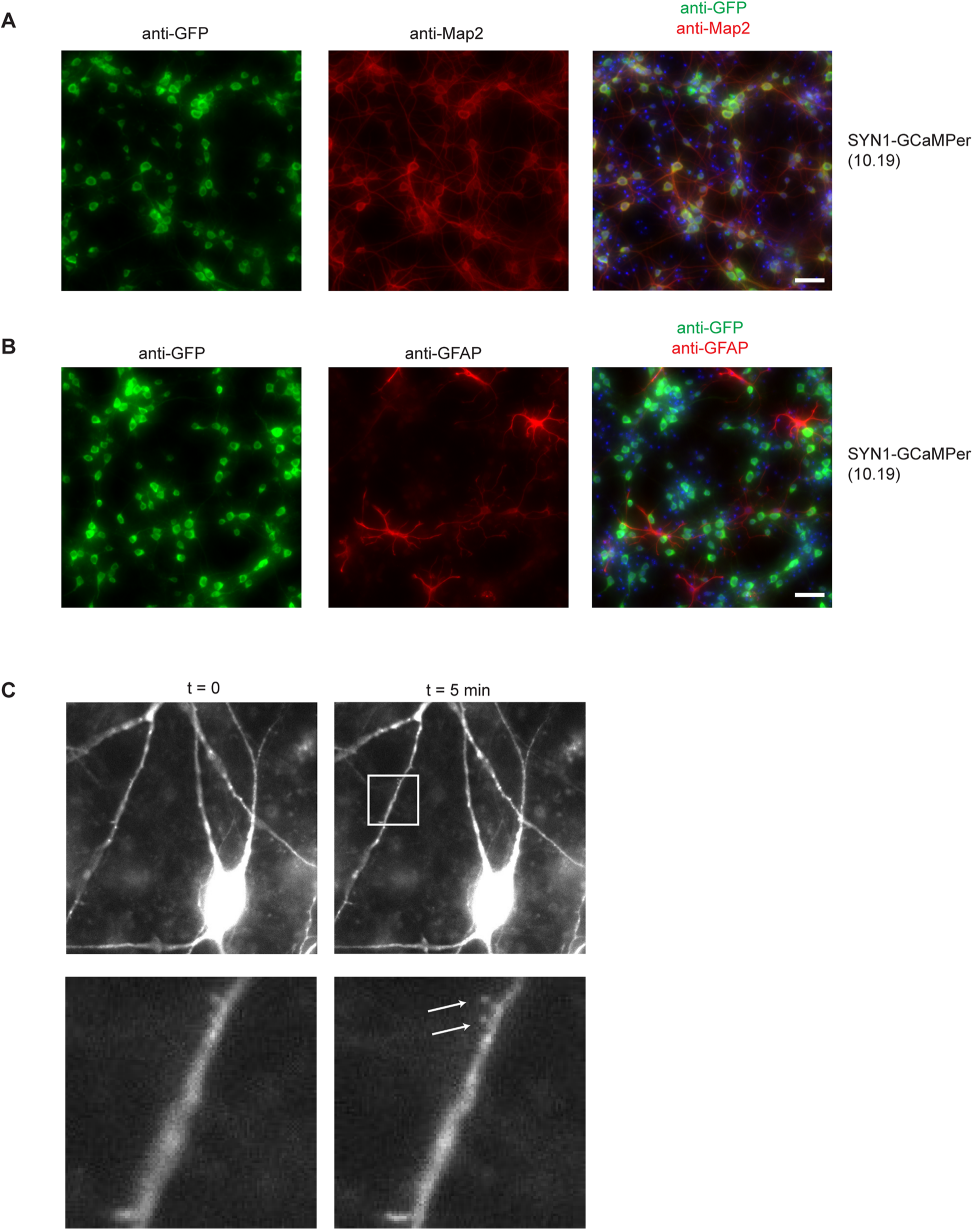
AAV-hSYN1-GCaMPer expression in rat primary cortical neurons. Rat primary cortical neurons were fixed 7 days after transducing with AAV-hSYN1-GCaMPer (10.19). Cells were immunostained with anti-GFP (green) and either the neuronal marker Map 2 (a) or astrocytic marker GFAP (b). Nuclei were stained with DAPI. Scale bar = 50 microns. (c) High magnification images of live primary cortical neurons expressing GCaMPer. Arrows indicate spine-like structures containing GCaMPer fluorescence.

We next tested GCaMPer (10.19) in rat primary cortical cultures. These cultures contain a mix of both neuronal and glial cell types. We constructed an AAV-vector containing the human synapsin 1 promoter (hSYN1), to restrict expression to neuronal cells [40]. After transducing cells with AAV-hSYN-1 GCaMPer (10.19), we detected expression in neuronal cells (Fig 3A), with no detectable expression in GFAP-positive astrocytic cells (Fig 3B). A majority of expression was observed in the neuronal soma (Fig 3A and 3B), but also detectable in the projections, where small spine-like structures were observed at high magnification (Fig 3C, S2 Video).

**Fig 4:**
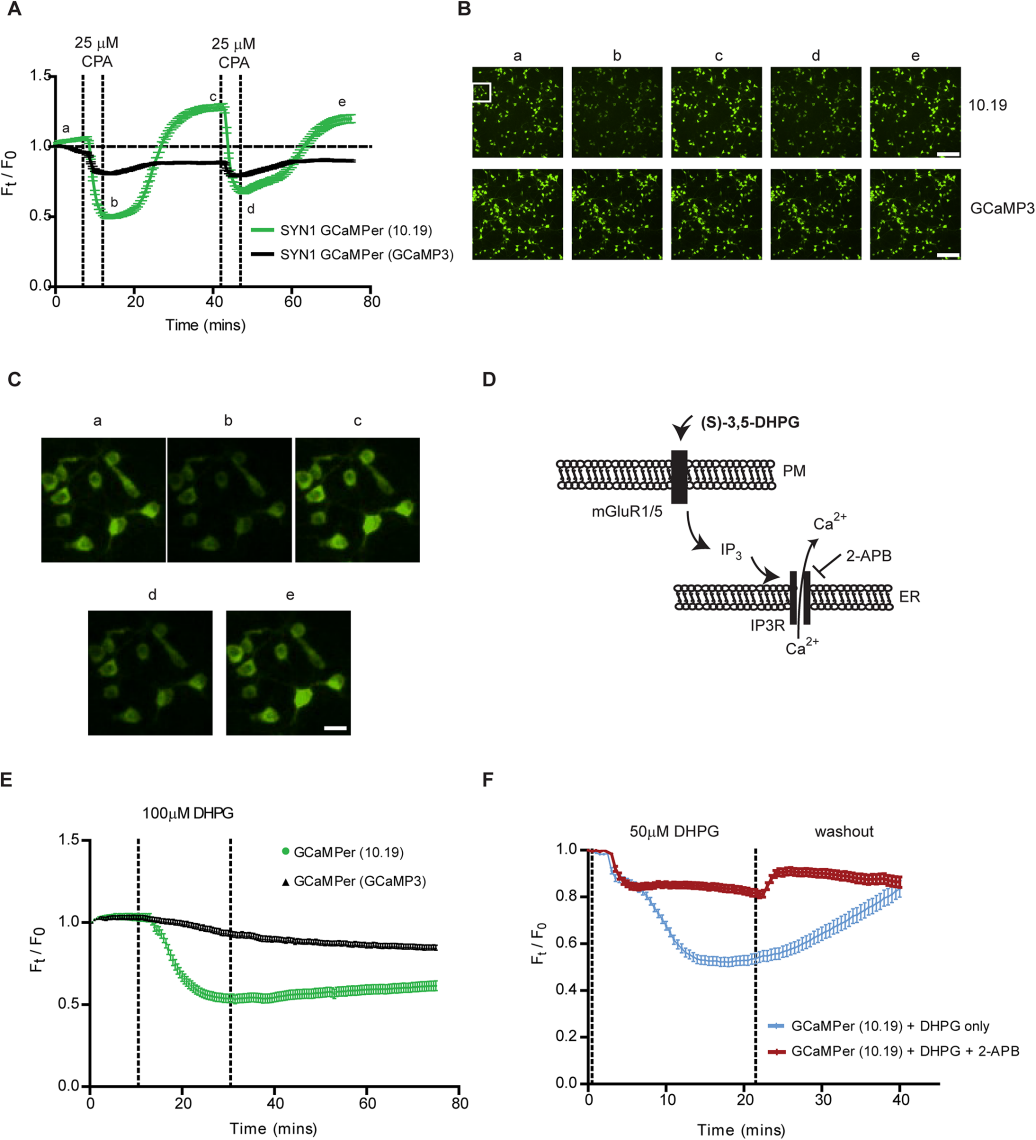
GCaMPer fluorescence is altered by reversible ER calcium store depletion. (a) Rat primary cortical neurons transduced with AAV-hSYN1-GCaMPer (10.19 or GCaMP3) were treated for two cycles with 25 μM CPA followed by 30 min washout of the SERCA inhibitor. Fluorescence was tracked in individual cells over the timecourse (mean ± SEM, n = 100 cells). (b) Micrographs at indicated timepoints in panel a. Scale bar = 200 microns. (c) High magnification view of GCaMPer (10.19)-expressing neurons. Field-of-view indicated by white box in panel b. Scale bar = 40 microns. (d) Diagram of approach to alter ER calcium stores by activating IP_3_Rs using (S)-3,5-DHPG. (e) Rat primary cortical neurons expressing GCaMPer (10.19 or GCaMP3) were treated with 100 μM DHPG to activate IP_3_Rs. Fluorescence of individual cells was measured every 30 sec over 80 min (mean ± SEM, n = 75 cells). (f) The contribution of IP_3_R activity in mediating DHPG-induced GCaMPer (10.19) fluorescence was examined using 2-APB to block IP_3_R efflux. Fluorescence of individual cells was measured every 30 sec over 40 min (mean ± SEM, n = 75 cells).

GCaMPer fluorescence was next assessed in rat primary cortical neurons exposed to pharmacologic modulators of the ER calcium store. Exposure to CPA caused a decrease in fluorescence that was reversible with washouts (Fig 4A–4C, S3 Video). Effects of neuronal signaling on the ER store were examined using a metabotropic glutamate receptor (mGluR) agonist, (S)-3,5-DHPG. This compound indirectly activates IP_3_R-mediated ER Ca^2+^ efflux through the generation of IP_3_(Fig 4D). Following treatment, a decrease in GCaMPer (10.19) fluorescence was observed (Fig 4E). As predicted, the effect of (S)-3,5,-DHPG could be blocked by co-administration of 2-APB, an IP_3_R antagonist (Fig 4F).

**Fig 5:**
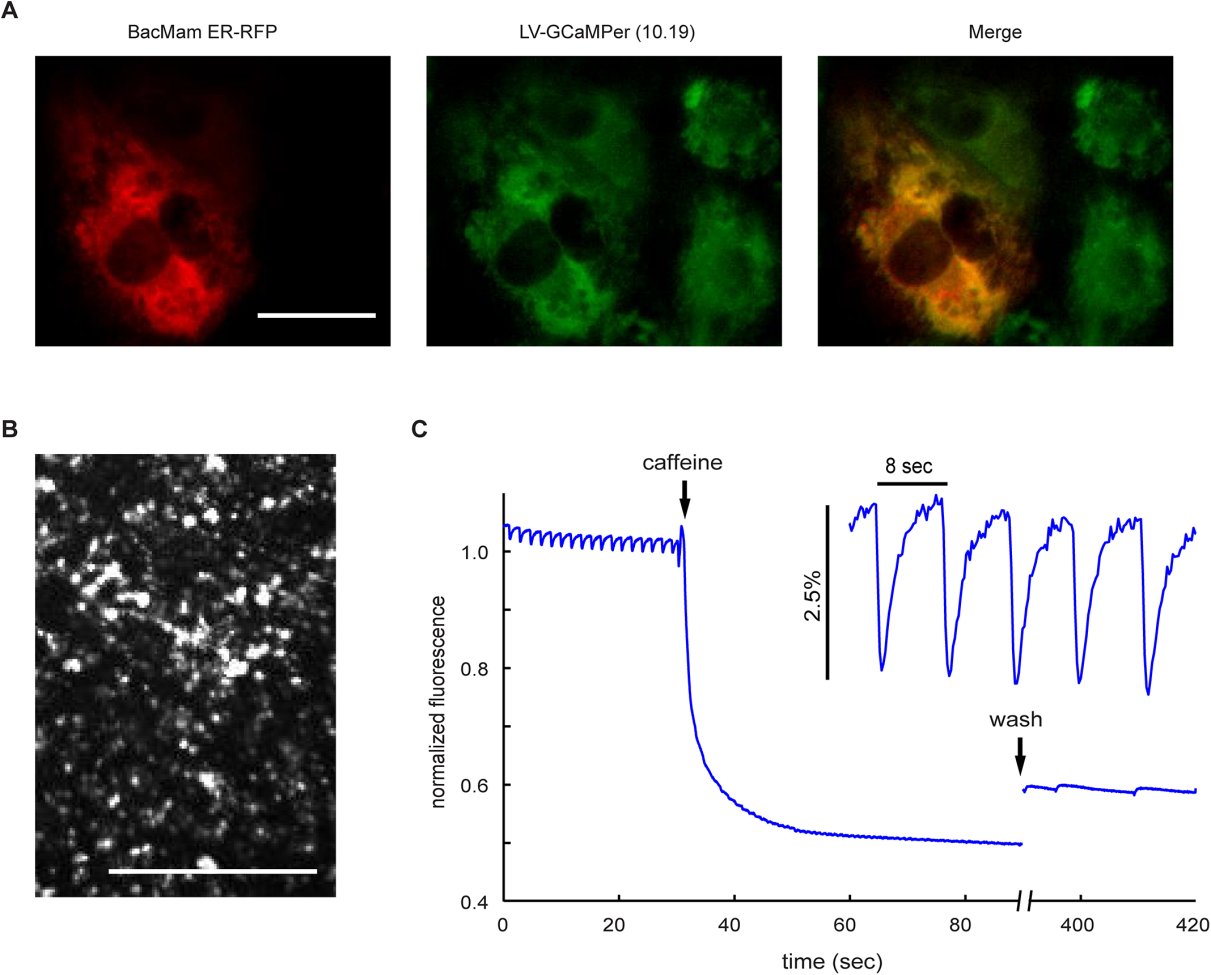
GCaMPer (10.19) to image changes in the ER calcium store during cardiomyocyte beating. (a) Human induced pluripotent stem cell-derived cardiomyocytes were transduced with BacMam ER RFP (red) and LV-GCaMPer (10.19) (green). Scale bar = 30 microns. (b) GCaMPer (10.19) expressing cardiomyocytes were examined under low magnification. Scale bar = 1 mm. (c) GCaMPer (10.19) fluorescence was captured (20 frames per second) during spontaneous beating. After 30 seconds, cells were perfused with 5 mM caffeine.

We next imaged the ER calcium store in beating cardiomyocytes using GCaMPer (10.19). We used human induced-pluripotent stem cell derived cardiomyocytes, which have emerged as a promising model for studying genetically based cardiac diseases as well as for probing drug-induced cardiotoxicity. Alterations in ER calcium handling are critical in modulating cardiac function. ER localization of GCaMPer (10.19) in the cardiomyocytes was confirmed by colocalization with an established ER-localized RFP (Fig 5A). Next, GCaMPer (10.19) fluorescence was examined during spontaneous beating using low-magnification microscopy (Fig 5B). The cardiomyocyte syncytium showed efflux from the ER to the cytosol at each beat with a ΔF/F_0_ of 2.5% (Fig 5C). The small fluorescence change is expected as only a small fraction of ER calcium is ejected to the cytosol during each beat. Perfusion with 5 mM caffeine stimulated ER Ca^2+^ release into the cytosol [41] and a rapid decrease in GCaMPer (10.19) fluorescence much larger in magnitude than that observed for a typical beat. Following caffeine addition, the calcium beats were abolished. After washing out the caffeine and allowing a five minute recovery period, the cell started beating again (albeit slowly and irregularly) and beat-associated decreases in ER calcium were detected again.

## Discussion

The use of genetically encoded calcium sensors has revolutionized the approach to understanding intracellular calcium dynamics. For example, GCaMP6 can be used to detect individual action potentials of specific neuronal populations in an intact animal [33]. Many of the GCaMPs used to date are optimized to monitor changes in cytoplasmic calcium, requiring a relatively high affinity for calcium (K_d_ = 100–500 nM) [33]. In the ER lumen, calcium can approach millimolar concentrations [4], which precludes use of the high affinity GECIs in the ER. Here we selected a low affinity (K_d_~ 400 μM) variant of GCaMP3 to create a GECI for the ER. This indicator demonstrates a decrease in fluorescence in response to ER calcium depletion.

The calcium binding affinities for GCaMP3 (10.19) were determined *in vitro*, using a recombinant protein that lacks the targeting sequences appended to GCaMPer (signal peptide and ER retention motif). The signal peptide is not expected to alter calcium binding, as it is predicted to be cleaved during ER translocation. The TAEKDEL sequence, however, remains appended to the mature protein, which could alter the affinity for calcium. The Ca^2+^ affinity of GCaMPer (10.19) was also measured in rat primary cortical neurons (Fig 1E), with an apparent K_d_ of 238 μM.

GCaMPer was localized primarily to the endoplasmic reticulum in the cell types examined, with little expression detected in the Golgi. In primary cortical neurons, a majority of GCaMPer fluorescence was observed in the soma. This localization could be due to an enrichment of ER in the soma, a lack of GCaMPer transport into peripheral regions of the cell, a gradient of ER calcium concentration in ER subdomains, or a combination thereof. Our ability to detect GCaMPer expression in neurites (Fig 3C) may allow for examination of ER calcium dynamics in neuronal substructures, such as spines.

A positive slope in GCaMPer (10.19) fluorescence (e.g. Fig 4A) was often observed at early experimental timepoints, coinciding with exchange into imaging medium. Optimizing the constituents of imaging medium or including a pre-equilibration phase at the start of GCaMPer experiments, to allow for cellular adaptation, may be beneficial before beginning experimental manipulations. Using pharmacologic approaches, we show changes in GCaMPer fluorescence that correspond to depleting the ER calcium store. Thapsigargin, a high affinity inhibitor of SERCA typically considered irreversible [42], caused a decrease in fluorescence that did not recover after washout. In contrast, recovery of GCaMPer fluorescence was observed with the lower affinity SERCA inhibitor CPA. Enhancing calcium efflux with 4-CmC or 3,5-DHPG reduced GCaMPer fluorescence in neurons, and caffeine had a comparable effect in cardiomyocytes.

While a decrease in GCaMPer fluorescence is indicative of a change in ER calcium content, there are alternate interpretations of this result. First, photobleaching of the fluorophore can occur, and a recent report indicates GFP photobleaching is more rapid in an oxidizing environment, as would be expected in the ER lumen [43]. Second, decreases in fluorescence may be related to loss of ER retention and subsequent secretion of the protein. Notably, secretion of BiP, a KDEL containing protein, has been observed in response to thapsigargin treatment [44]. To control for these potential artifacts, we examined GCaMPer (GCaMP3) with the same N- and C-terminal modifications. We anticipate that this protein will not show calcium-dependent dynamics in the ER, but will be subject to similar photobleaching and transport effects. GCaMPer (GCaMP3) showed a moderate decrease in fluorescence over time, likely due to photobleaching, but did not show the dramatic pharmacological responses observed for GCaMPer (10.19). The recovery of GCaMPer (10.19) fluorescence after drug washout is clear evidence of ER calcium sensing, as is the beat-associated downward transient observed in cardiomyocytes.

While we were developing GCaMPer, Suzuki *et al*. reported G-CEPIA1er for monitoring changes in ER calcium [29]. GCaMPer and G-CEPIA1er contain a different group of amino acid substitutions, but are similar in their reduced affinities for calcium (K_d_ = 400 μM versus K_d_ = 672 μM *in vitro*) and responses to CPA in SH-SY5Y cells (Fig 2F). The proteins are targeted to the ER via a similar approach, but using a different set of amino- and carboxy-terminal sequences. G-CEPIA1er utilizes the signal peptide from Ig heavy chain, whereas GCaMPer contains the signal peptide from calreticulin. G-CEPIA1er uses SEKDEL (rodent GRP78/BiP) as the ER retention motif versus TAEKDEL (human GRP78/BiP) for GCaMPer. The serine residue at the minus six position was identified as a phosphorylation site in mouse liver [45]; however, the importance of the residue for ER retention is not characterized. Owing to reports demonstrating the importance of positions -5 and -6 on the ability of a KDEL sequence to retain proteins in the ER [46], as well as KDEL receptor isoform specificity in the retention process [47], it is possible that the retention efficiency for GCaMPer and G-CEPIA1er will differ between cell types.

Tools to monitor ER calcium are important for understanding basic cellular biology and to characterize pathologies associated with disruption of this calcium store. Overall, GCaMPer (10.19) demonstrates responsiveness to ER calcium flux, exhibits a good signal-to-noise ratio, and extends the versatility of genetically encoded calcium sensors for real-time monitoring of the ER calcium store.

## Supporting Information

S1 Fig Plasmids used in this study. Addgene plasmid numbers are indicated.
(PDF)

S1 Video GCaMPer (10.19) fluorescence was monitored in SH-SY5Y cells exposed to multiple rounds of reversible SERCA inhibition using cyclopiazonic acid. Time is indicated in the upper left corner and compound additions in the lower right corner. This video is associated with data presented in Fig 2.
(AVI)

S2 Video Rat primary cortical neurons were transduced with AAV-hSYN1-GCaMPer (10.19) and examined by high magnification microscopy to visualize small spine-like structures. This video is associated with data presented in Fig 3.
(AVI)

S3 Video Rat primary cortical neurons were transduced with AAV-hSYN1-GCaMPer (10.19) and exposed to multiple rounds of reversible SERCA inhibition using cyclopiazonic acid. Time is indicated in the upper left corner and compound additions in the lower right corner. This video is associated with data presented in Fig 4.
(AVI)

## Abbreviations

ER: endoplasmic reticulum
SERCA: sarco/endoplasmic reticulum calcium ATPase
RyR: ryanodine receptor
IP_3_R: inositol triphosphate receptor
SR: sarcoplasmic reticulum
GECI: genetically-encoded calcium indicator
GCaMPer: ER-targeted GCaMP indicator protein
CEPIA1er: calcium-measuring organelle-entrapped protein indicator 1 in the ER
WT: wild-type
DIV: day in vitro
hiPSC-CMs: human induced pluripotent stem cell-derived cardiomyocytes
AAV: adeno-associated virus
LV: lentivirus
