# Supplementary material for: A Low Affinity GCaMP3 Variant (GCaMPer) for Imaging the Endoplasmic Reticulum Calcium Store
Source: PLoS One. 2015 Oct 9;10(10):e0139273. doi: 10.1371/journal.pone.0139273 (PMC4599735; doi:10.1371/journal.pone.0139273)
Supplement: S1 Fig — Addgene plasmid numbers are indicated. (PDF) [file pone.0139273.s001.pdf]

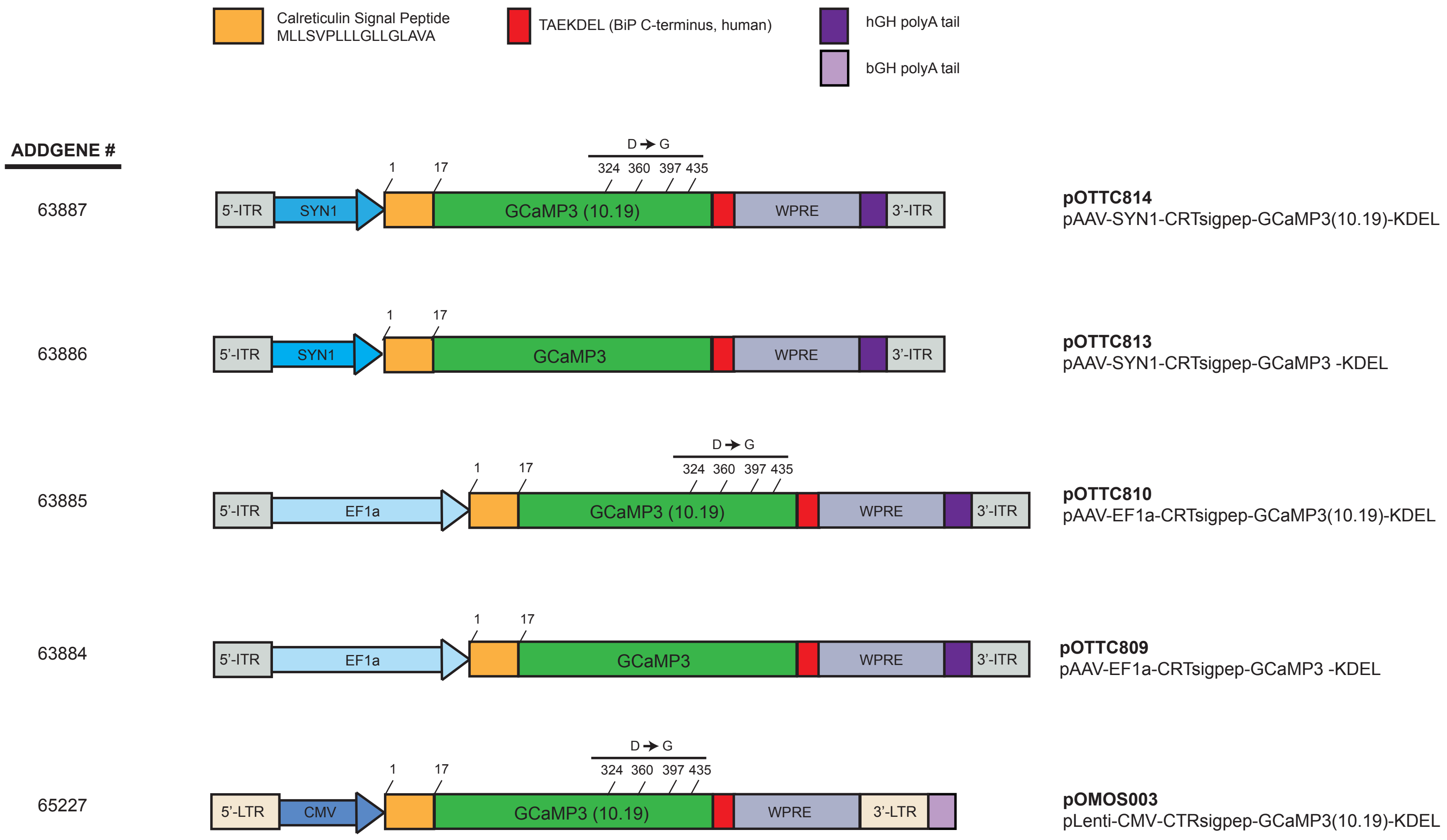
Calreticulin Signal Peptide
MLLSVPLLLGLLGLAVA
TAEKDEL (BiP C-terminus, human)
hGH polyA tail
bGH polyA tail
ADDGENE #
63887
5'-ITR SYN1 GCaMP3 (10.19) WPRE 3'-ITR
1 17 D → G 324 360 397 435
pOTTC814
pAAV-SYN1-CRTsigpep-GCaMP3(10.19)-KDEL
63886
5'-ITR SYN1 GCaMP3 WPRE 3'-ITR
1 17
pOTTC813
pAAV-SYN1-CRTsigpep-GCaMP3 -KDEL
63885
5'-ITR EF1a GCaMP3 (10.19) WPRE 3'-ITR
1 17 D → G 324 360 397 435
pOTTC810
pAAV-EF1a-CRTsigpep-GCaMP3(10.19)-KDEL
63884
5'-ITR EF1a GCaMP3 WPRE 3'-ITR
1 17
pOTTC809
pAAV-EF1a-CRTsigpep-GCaMP3 -KDEL
65227
5'-LTR CMV GCaMP3 (10.19) WPRE 3'-LTR
1 17 D → G 324 360 397 435
pOMOS003
pLenti-CMV-CTRsigpep-GCaMP3(10.19)-KDEL
